# Mood Disturbances in a Patient on Statin Therapy: A Case Report

**DOI:** 10.7759/cureus.89670

**Published:** 2025-08-09

**Authors:** Rahma Nazar, Dabeet Sajeev

**Affiliations:** 1 General Medicine, Maidstone and Tunbridge Wells NHS Trust, Kent, GBR; 2 General Internal Medicine, Royal Derby Hospital, Derby, GBR

**Keywords:** adverse drug reaction, cholesterol and mood, mood disturbances, neuropsychiatric effects, statin intolerance

## Abstract

Statins (3-hydroxy-3-methylglutaryl coenzyme A reductase inhibitors) are widely used to treat hyperlipidaemia and significantly reduce the risk of cardiovascular events. While generally well tolerated, emerging evidence suggests that statins may, in rare cases, be associated with neuropsychiatric adverse effects, including mood disturbances and behavioural changes. We report the case of a 54-year-old woman with hypertension, but no personal or family history of psychiatric illness, who developed mood and behavioural changes shortly after initiating atorvastatin 10 mg following the detection of dyslipidaemia on routine blood testing. Her regular medications included bisoprolol and amlodipine, which she had been taking for eight years without any prior neuropsychiatric side effects. Symptoms resolved rapidly upon discontinuation and recurred upon rechallenge, prompting cessation and referral for psychological counselling. The Naranjo Adverse Drug Reaction (ADR) Probability Scale yielded a score of 8, indicating a probable relationship between the statin and the mood disturbances. Although uncommon, statin-induced neuropsychiatric symptoms have been documented in genetic studies and pharmacovigilance databases. Proposed mechanisms include reduced central nervous system cholesterol affecting serotonergic neurotransmission and statin-related modulation of cytokine signalling. This case highlights the importance of clinician awareness of potential mood-related side effects of statins, even in patients without prior psychiatric history or those receiving low doses. Early recognition and timely management can minimise patient distress and support safer long-term adherence to lipid-lowering therapy.

## Introduction

Statins (3-hydroxy-3-methylglutaryl coenzyme A reductase inhibitors) are widely used to treat hyperlipidaemia and significantly reduce the risk of cardiovascular events. They are among the best-selling classes of prescription medications worldwide [1]. While generally well tolerated, statins have been associated with a range of neuropsychiatric adverse effects, including mood disturbances, depression, and even suicidal ideation, documented in pharmacovigilance databases [2] and genetic studies [3].

The relationship between serum cholesterol and mood regulation is complex and controversial, with inconsistent findings across epidemiological and clinical studies [4]. It has been suggested that low serum cholesterol may disrupt cerebral serotonin metabolism, which plays a key role in mood regulation [5], although this remains one of several proposed mechanisms for the potential neuropsychiatric effects of statins. Other hypotheses include statin-induced alterations in cytokine signalling and inflammatory pathways [6,7], as well as mitochondrial dysfunction triggered by oxidative stress [8,9]. Mitochondria are essential for neuronal energy metabolism, and their impairment may contribute to neurotoxicity and behavioural changes.

The ability of statins to cross the blood-brain barrier may further contribute to their potential neuropsychiatric effects. Lipophilic statins demonstrate greater central nervous system (CNS) penetration [10,11], though even hydrophilic statins may exert indirect effects. Additionally, statin-induced oxidative stress may increase blood-brain barrier permeability [12,13], allowing greater CNS drug exposure. While direct evidence linking statins to psychological symptoms is limited, we present a case in which mood-related symptoms appeared after initiating statin therapy and resolved with its discontinuation.

## Case presentation

A 54-year-old woman with a known history of hypertension and no personal or family history of psychiatric illness underwent routine blood investigations, which revealed dyslipidaemia, characterised by elevated total cholesterol and low-density lipoprotein (LDL), moderately increased triglycerides, and markedly reduced high-density lipoprotein (HDL) (Table 1). Physical examination was unremarkable, with stable vital signs and no neurological or psychiatric abnormalities observed at baseline. Her regular medications included bisoprolol 2.5 mg and amlodipine 2.5 mg daily, both of which she had been taking for eight years without any prior neuropsychiatric effects.

**Table 1:** Lipid profile at baseline and after five weeks of statin treatment HDL-C: high-density lipoprotein cholesterol, LDL-C: low-density lipoprotein cholesterol, M: male, F: female.

Laboratory parameter	Baseline	After five weeks of statin therapy	Reference range
Total cholesterol	222 mg/dL	183 mg/dL	<193 mg/dL
HDL-C	18 mg/dL	40 mg/dL	>39 mg/dL (M); >46 mg/dL (F)
LDL-C	162 mg/dL	110 mg/dL	<116 mg/dL
Non-fasting triglyceride	210 mg/dL	165 mg/dL	<177 mg/dL

Given her cardiovascular risk profile, she was initiated on atorvastatin 10 mg once daily as the primary prevention. Within three weeks of starting statin therapy, she began exhibiting mood and behavioural changes that were markedly out of character. These included persistent irritability, increased impatience, and a heightened temper, particularly toward family members. She did not report depressive thoughts, anxiety, hallucinations, or sleep disturbances.

Comprehensive blood workup was performed to exclude organic causes of mood disturbance, including serum electrolytes, thyroid function tests, and liver and renal profiles, all of which were within normal ranges.

Despite improvement in her lipid profile after five weeks, she discontinued atorvastatin due to persistent psychological symptoms. Notably, her mood improved significantly within two days of discontinuation, and she was able to resume her normal daily activities.

Six months later, repeat lipid testing showed a rise in total cholesterol, leading to re-initiation of atorvastatin at the same dose. Within one week, the patient developed a recurrence of neuropsychiatric symptoms, this time more severe, manifesting as tearfulness, apathy, and social withdrawal. Once again, she discontinued the statin, leading to the rapid resolution of symptoms. She also began psychological counselling with a mental health professional, focusing on emotional support and coping strategies, although no formal psychiatric diagnosis was made. Both the patient and her family reported a return to her baseline personality within days of stopping the medication.

## Discussion

Neuropsychiatric effects of statins, although uncommon, have been documented in the literature, ranging from mild symptoms to more severe outcomes, including depression and suicidal ideation. While large-scale randomised controlled trials have not consistently demonstrated increased psychiatric risk, individual case reports and observational studies suggest that a subset of patients may be vulnerable to such adverse effects.

**Figure 1:**
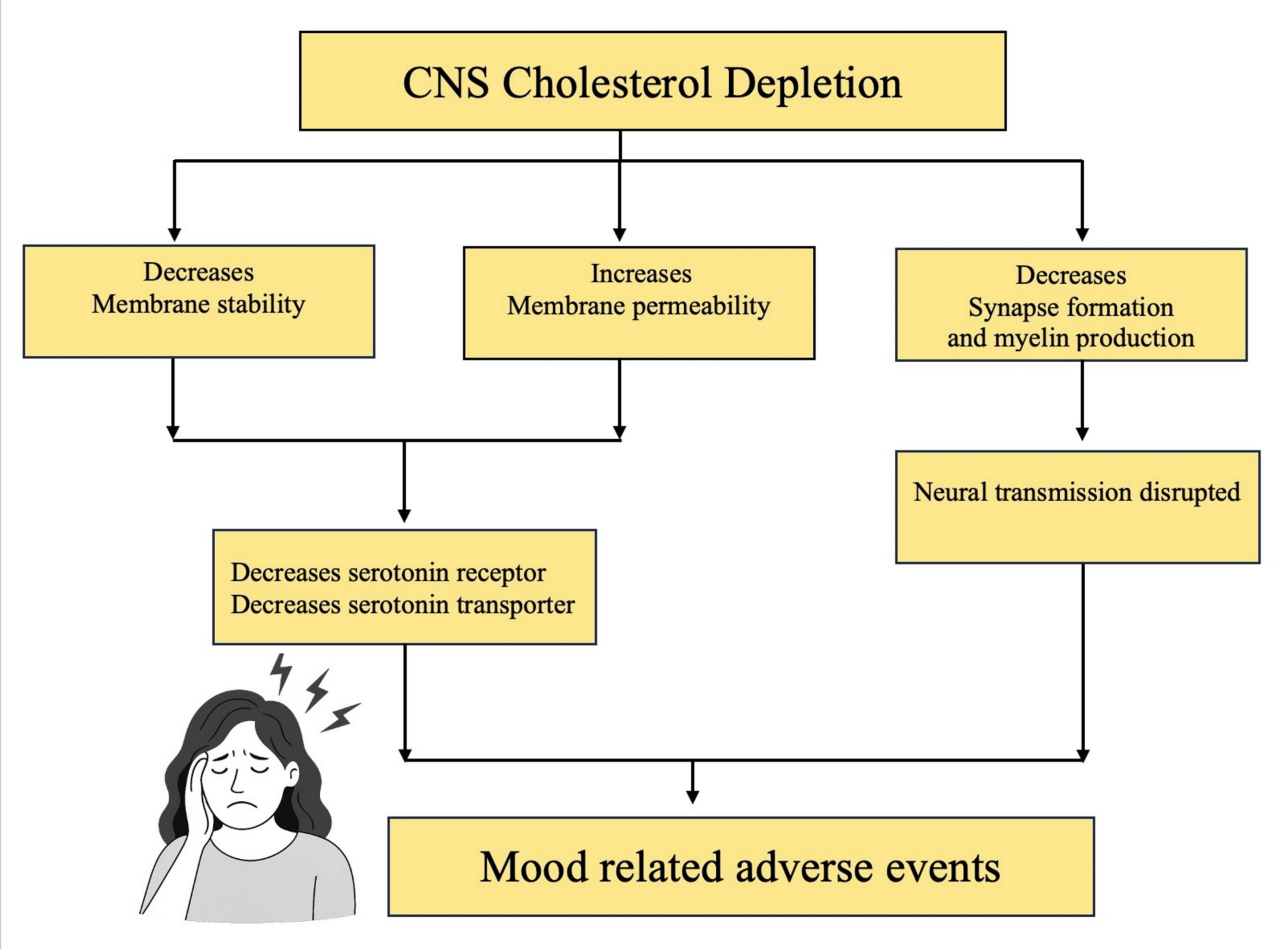
Pathophysiological cascade from CNS cholesterol depletion to mood disturbance Image Credits: Rahma Nazar. Information adapted from reference [14].

Several hypotheses have been proposed to explain these phenomena. One involves the depletion of CNS cholesterol (Figure 1). Cholesterol plays a critical role in maintaining membrane stability, reducing permeability, and modulating serotonergic function. Lower cholesterol levels may impair the function of serotonin receptors such as 5-HT1A and 5-HT7, as well as serotonin transporter activity. Beyond serotonin, cholesterol is essential for synapse formation and myelin production, and its reduction may have broader effects on neurotransmission, influencing gamma-aminobutyric acid (GABA) receptors, N-methyl-D-aspartate (NMDA) receptors, opioid signalling, and the transport of excitatory amino acids [14].

A second mechanism relates to the anti-inflammatory and immunomodulatory effects of statins. These drugs downregulate inflammatory cytokines, such as IL-6 and TNF-α, which are paradoxically implicated in the pathogenesis of both atherosclerosis and mood disorders [6,7]. The imbalance in cytokine signalling may thus induce depressive symptoms through altered neuroimmune interactions.

Statin-induced oxidative stress and mitochondrial dysfunction have been increasingly recognised. Mitochondria play a crucial role in neuronal energy metabolism, and oxidative stress can impair their function, potentially causing neurotoxicity and behavioural changes. Some studies suggest that the pathophysiological mechanisms involved in statin-induced myopathy, another well-known side effect, may also contribute to neuropsychiatric symptoms, supporting the concept of systemic vulnerability [9,15].

Lipophilic statins, such as simvastatin and atorvastatin, demonstrate greater penetration across the blood-brain barrier compared to their hydrophilic counterparts [10,11], although all statins retain some ability to access the CNS. The clinical relevance of this enhanced CNS entry remains uncertain, as peripheral statin effects, such as changes in cytokine signalling or lipid metabolism, may also produce central consequences. Furthermore, statin-induced oxidative stress has been shown to increase blood-brain barrier permeability, potentially enhancing CNS exposure to the drug [13]. Interestingly, most reported cases of neuropsychiatric adverse events involve lipophilic statins; however, these agents also represent the most commonly prescribed statins historically [16], which may contribute to their overrepresentation in the literature.

While the patient's concomitant medication, bisoprolol, has been associated with rare neuropsychiatric adverse effects such as fatigue, depression, and sleep disturbances, she had been on a stable low dose for eight years without any prior symptoms, making it an unlikely contributor to the mood changes observed.

In this case, psychiatric symptoms varied from irritability to apathy and appeared following statin use. Notably, the patient had no prior psychiatric history and was on a relatively low dose of atorvastatin, yet developed significant mood changes shortly after initiation of therapy. The rapid emergence of symptoms following statin initiation, along with their recurrence upon reintroduction, highlights the importance of recognising individual variability in susceptibility and the need for careful monitoring of mood changes, even in patients without known risk factors.

The Naranjo Adverse Drug Reaction (ADR) Probability Scale was used to assess causality, which yielded a score of 8 (Table 2). This suggests a probable relationship between statin therapy and mood disturbances, given the reasonable temporal sequence, the known response profile to the drug, symptom resolution on withdrawal, and absence of alternative explanations related to the patient’s clinical status [17].

**Table 2:** The Naranjo Algorithm The Naranjo Algorithm is a questionnaire for determining the likelihood of whether an adverse drug reaction (ADR) is actually due to the drug rather than the result of other factors. The patient scored 8 on the algorithm, demonstrating a probable adverse drug reaction. Information adapted from reference [18]. This material is in the public domain and used with permission from the LiverTox editorial staff.

Question	Yes	No	Do not know	Score
Are there previous conclusive reports on this reaction?	1	0	0	1
Did the adverse event appear after the suspected drug was given?	2	-1	0	2
Did the adverse reaction improve when the drug was discontinued or a specific antagonist was given?	1	0	0	1
Did the adverse reaction appear when the drug was re-administered?	2	-1	0	2
Are there alternative causes that could have caused the reaction?	-1	2	0	2
Did the reaction reappear when a placebo was given?	-1	1	0	0
Was the drug detected in any body fluid in toxic concentrations?	1	0	0	0
Was the reaction more severe when the dose was increased, or less severe when the dose was decreased?	1	0	0	0
Did the patient have a similar reaction to the same or similar drugs in any previous exposure?	1	0	0	0
Was the adverse event confirmed by any objective evidence?	1	0	0	0
				Total score: 8

 Table 3 shows the scoring interpretation of the Naranjo Algorithm.

**Table 3:** Scoring interpretation of the Naranjo Algorithm ADR: adverse drug reaction. Information adapted from reference [18]. This material is in the public domain and used with permission from the LiverTox editorial staff.

Total score	Interpretation of scores
≥9	Definite ADR. The reaction (1) followed a reasonable temporal sequence after a drug or in which a toxic drug level had been established in body fluids or tissues, (2) followed a recognised response to the suspected drug, and (3) was confirmed by improvement on withdrawing the drug and reappeared on re-exposure.
5-8	Probable ADR. The reaction (1) followed a reasonable temporal sequence after a drug, (2) followed a recognised response to the suspected drug, (3) was confirmed by withdrawal but not by exposure to the drug, and (4) could not be reasonably explained by the known characteristics of the patient’s clinical state.
1-4	Possible ADR. The reaction (1) followed a temporal sequence after a drug, (2) possibly followed a recognised pattern to the suspected drug, and (3) could be explained by characteristics of the patient’s disease.
≤0	Doubtful ADR. The reaction was likely related to factors other than a drug.

However, as with all case reports, causality cannot be definitively established. While the pattern of symptom recurrence and resolution is suggestive, the findings should be interpreted with caution. In this case, statin therapy was discontinued entirely, and although alternative options such as switching to a hydrophilic statin were considered, these were not pursued. Follow-up after psychological counselling revealed resolution of mood symptoms and a return to the patient’s baseline mental state. Further large-scale observational and interventional studies are needed to better characterise any potential neuropsychiatric effects of statins and the mechanisms that may underlie them.

## Conclusions

This case highlights an important consideration regarding the psychiatric safety profile of statins. While their cardiovascular benefits are well established, clinicians should remain vigilant for potential mood changes, particularly in patients newly initiated on therapy, as early recognition and appropriate management can prevent significant distress and improve patient outcomes. The recurrence of symptoms upon re-exposure in this patient further supports a potential causal relationship, emphasising the need for personalised treatment approaches. Clinicians should consider the possibility of neuropsychiatric side effects when evaluating unexplained behavioural changes in otherwise stable individuals. Further research is warranted to better understand the underlying mechanisms and to identify patients who may be at increased risk.
